# Streptococci in the Subgingival Biofilm and Periodontal Therapy

**DOI:** 10.3290/j.ohpd.b875517

**Published:** 2021-01-26

**Authors:** Holger Jentsch, Arne C. Rodloff, Maria Kristina Gerweck, Catalina-Suzana Stingu

**Affiliations:** a Centre for Periodontology, Clinic of Conservative Dentistry and Periodontology, University of Leipzig Medical Centre, Leipzig, Germany. Study concept, data analysis and wrote the paper, revised the manuscript and gave final approval for publication.; b Professor Doctor, Institute for Medical Microbiology and Epidemiology of Infections, University of Leipzig Medical Centre, Leipzig, Germany. Study concept, revised the manuscript and gave final approval for publication.; c Doctor of Dentistry, Centre for Periodontology, Department for Cariology, Endodontology and Periodontology, University Hospital Leipzig AöR Leipzig, Germany. Data collection, revised the manuscript and gave final approval for publication.; d Assistant Professor, Institute for Medical Microbiology and Epidemiology of Infectious Diseases, University Hospital of Leipzig AöR, Leipzig, Germany. Study concept, data analysis and wrote the paper, revised the manuscript and gave final approval for publication.

**Keywords:** periodontitis, professional dental prophylaxis, subgingival instrumentation, subgingival biofilm, viridans-streptococci

## Abstract

**Purpose:**

The aim of this study was to verify how the prevalence of viridans-streptococci is changed by two appointments of professional prophylaxis and after the subgingival instrumentation via scaling and root planing (SRP).

**Material and Methods:**

Samples of the subgingival biofilm were collected from 19 individuals with periodontitis receiving two appointments of professional prophylaxis and SRP before and after the treatment procedures and the presence of viridans-streptococci was analysed by microbiological cultivation. Non-parametric statistical testing using Friedman/Wilcoxon tests and chi-square testing was used for statistical analysis.

**Results:**

No statistically significant changes over time were found for the mutans-group. The prevalence of *Streptococcus mitis* decreased after two appointments of professional prophylaxis (p = 0.013). The prevalence of *S. mitis* decreased again after SRP (p <0.001). The prevalence of *Streptococcus anginosus* decreased after two appointments of professional prophylaxis (p = 0.002). After SRP five positive results for *S. anginosus* were detected (p = 0.026). For *Streptococcus oralis* and *Streptococcus gordonii* tendencies to statistical significance were found. The number of positive results for *S. oralis* increased after the first appointment of professional oral prophylaxis (p = 0.055). The number of positive results for *S. gordonii* increased after the first appointment of professional oral prophylaxis (p = 0.055).

**Conclusion:**

The step-wise periodontal therapy influences the prevalence of viridans-streptococci, especially *S. mitis* and *S. anginosus*. No tremendous increase of streptococci especially related to the carious process occurs in the subgingival biofilm.

**Clinical Relevance:**

The study reveals knowledge on changes of the composition of the subgingival biofilm due to different steps of periodontal therapy.

Periodontitis is not just a simple bacterial infection but a complex multifactorial disease.^28^ Nevertheless the composition of the subgingival biofilm is of outstanding importance, it decides between health and disease or coexistence without tissue damage and initiation and continuation of periodontal destruction, respectively. Respecting the keystone hypothesis, keystone pathogenic microorganisms initiate the inflammatory process and lead to dysbiosis by disturbing the homeostatic balance.^21^ *Porphyromonas gingivalis* is one of the most important keystone pathogens, is frequently detected in the microbiota of periodontally healthy individuals and is on the other hand strongly related to periodontal tissue damage.^11,12^ The amount of *P. gingivalis* in the subgingival biofilm is reduced by subgingival instrumentation.^23^ The persistence of *P. gingivalis* after non-surgical periodontal therapy decides about the long-lasting therapeutical effect of scaling and root planning (SRP).^15^ On the other hand SRP itself reduced also significantly the proportion of anaerobe bacteria in the subgingival biofilm for up to more than 6 months.^39^

There is reduced knowledge how the composition of the subgingival biofilm improves by mechanical instrumentation alone. Many studies focused more or less on anaerobic periodontopathogenic bacteria and did not point out aspects of eubiosis such as the changes within the oral streptococci groups.^34^ The ability to colonise multiple oral surfaces and the production of inhibitory substances make oral streptococci key players in oral homeostasis and disease.^1^ Invernici et al described slight numeric enhancements of streptococci, especially of *Streptococcus oralis* 90 days after scaling and root planing (SRP) and more expressed in deeper periodontal pockets.^22^ Weekly repeated supragingival plaque removal after scaling and root planing for 3 months further reduced several subgingival species including streptococci species.^16,42^ The results were maintained up to 1 year and the authors found no major difference in the composition of the subgingival biofilm after the treatment process and samples from periodontally healthy individuals. An extensive search in the literature did not detect quantitative results on viridans-streptococci in the subgingival biofilm of periodontitis patients in the initial therapy phase where combined supragingival professional plaque removal and causative anti-infective and anti-inflammatory treatment via SRP is mandatory.^35^ Interactions among streptococcal species and their environment have shaped these bacteria toward carbohydrate catabolism and environmental acidification. Consequently health or disease status are often dependent on their metabolic activities.^1^ Therefore, the aim of this study was to verify how the prevalence of viridans-streptococci at species and group levels is changed by two appointments of professional prophylaxis and after the subgingival instrumentation via SRP. The hypothesis of the study was that different changes of prevalence of viridans-streptococci (increase of streptococci related more to the carious process, decrease of streptococci related more to periodontal inflammation) occur in the subgingival biofilm after the treatment procedures.^4,8,9,18,20,29,36^

## Materials and Methods

### Study Population

Following the approval of the study by the ethics committee of the Medical Faculty of the University of Leipzig, Germany (101-2005), 19 randomly selected patients were included in the study after screening and being asked to participate. All 19 patients gave written informed consent to their participation. The study was performed in the Centre for Periodontology of the Clinic of Conservative Dentistry and Periodontology, University of Leipzig Medical Centre, Germany, and respected the principles outlined in the Declaration of Helsinki, as revised in 2008. To be included in the study, the patients had to meet the following criteria: diagnosis of moderate to severe periodontitis, no periodontal treatment prior to the study, no antibiotic treatment within the last 3 months prior to the study, no diseases with relationship to periodontitis, more than 15 natural teeth and at least four periodontal pockets with a probing depth of ≥ 4 mm. Pregnancy and lactation as well as the need of periodontal surgery or antibiotics during the SRP were criteria for exclusion.

Examination and treatment procedures were executed by different clinicians. For intraexaminer calibration, repeated measurements of two quadrants in seven patients were executed and resulted in κ = 0.89.

### Therapy and Follow-Up Treatment

In the untreated patients two appointments of initial prophylaxis were performed followed by full-mouth scaling and root planing within 24 h. The treatment procedures were performed every two weeks. Hand and ultrasonic instruments were used for the appointments of professional prophylaxis and for the SRP. The SRP was performed under local anaesthesia with articaine hydrochloride (Ultracain D-S, Sanofi-Aventis, Frankfurt/Main, Germany). After the SRP all patients used chlorhexidine digluconate as a mouthrinse for 1 min twice daily for 7 days (Chlorhexamed forte 0.2%, GlaxoSmithKline, Bühl, Germany). No antibiotics were used, but instructed individual oral hygiene procedures using toothbrush and interdental brushes were applied.

### Clinical Variables and Sampling Procedures

Clinical variables were measured immediately before the first appointment of professional prophylaxis. The clinical variables probing depth (PD), attachment level (AL) and bleeding on probing (BOP) were recorded as a six-point measurement. The interproximal plaque index (API) was measured immediately before the first and second appointment of professional prophylaxis, immediately before the SRP and 2 weeks after the SRP. During the same appointments after registering the API the sampling of the subgingival biofilm was performed. For sampling the subgingival biofilm at the site with the highest PD per quadrant, sterile endodontic paper points (ISO 60, Roeko, Langenau, Germany) were inserted into the pocket until resistance was perceptible and were left in place for 30 seconds. After the removal out of the periodontal pockets the paper points were placed in 2 ml brain-heart-infusion bouillon and referred to the Institute for Medical Microbiology and Epidemiology of Infections within 2 h.

### Cultivation and Detection of Streptococci

The samples were immediately analysed. Broth dilutions (10^-1^, 10^-2^, 10^-3^) were cultured on Columbia blood agar (Thermo Fisher Scientific, Oxoid Microbiology Products, Hampshire, UK) for 48 h at 37ºC. All alpha or non-haemolytic, catalase-negative colonies were biochemically identified using the Rapid ID 32 STREP system (bioMérieux, Lyon, France) according to the manufacturer’s instructions.

### Data Analysis

The statistical program SPSS 18.0.2. was used to perform the statistical analyses. Four categories of the interproximal plaque index (< 25% = optimal, 26–41% = good, 42–70% = moderate, > 70% = poor) were used to determine if the presence of different strains of streptococci depends on supragingival plaque. Two groups based on the PD measurements were formed (< 6 mm, ≥6 mm) to determine if the presence of different strains of streptococci depends on probing pocket depth. Non-parametric statistical testing using Friedman/Wilcoxon tests and chi-square testing to compare frequencies was performed. A p value of ≤ 0.050 was set as statistically significant. A p value 0.05 < p < 0.1 was considered as a tendency to statistical significance.

## Results

The study was performed from June 2006 until June 2008. Fifty-eight patients were assessed for eligibility, 39 patients were excluded or refused to participate in the study. The demographic and clinical data of the 19 included patients are summarised in Tables 1 and 2. No severe adverse effects of the treatment procedures were observed during the study.

**Table 1:** Demographic data

Number of participants	n	19
**Age**	x–	59.3
**(years)**	s	11.84
**Sex**	male	7
	female	12
**Smoker**	yes	8
	no	11

**Table 2:** Clinical data

Variable	x– ± s
API (%) Before treatment After first prophylaxis After second prophylaxis	63.4 ± 26.4 33.3 ± 15.7 35.9 ± 15.3
PD (mm) before treatment at four sites of sampling biofilm	5.86 ± 1.97
AL (mm) before treatment at four sites of sampling biofilm	6.12 ± 2.08
BOP (%) before treatment at four sites of sampling biofilm	71.05 ± 19.35

The prevalence of different species, respectively, groups of streptococci, are presented in the Tables 3 and 4. The prevalence of two of the 13 streptococci species, *S. mitis* and *S. anginosus*, were significantly different between several appointments. *S. mitis* decreased after two appointments of professional prophylaxis from 37 to 23 positive results (p = 0.013) and decreased again after SRP from 23 to six positive results (p < 0.001). The prevalence of *S. anginosus* decreased after two appointments of professional prophylaxis from 15 to three positive results (p = 0.002). After SRP five positive results for *S. anginosus* were detected (p = 0.026). For *S. oralis* and *S. gordonii* tendencies to statistical significance were found. The number of positive results for *S. oralis* increased from 62 to 70 after the first appointment of professional oral prophylaxis (p = 0.055). The number of positive results for *S. gordonii* increased from 6 to 14 after the first appointment of professional oral prophylaxis (p = 0.055). After SRP 14 sites were positive for *S. gordonii* (p = 0.052).

**Table 3:** Prevalence of streptococci species at different appointments and statistical analysis

Streptococci species		before 1. prophylaxis	before 2. prophylaxis	before 1. prophylaxis	after 2. prophylaxis	before 1. prophylaxis	after SRP	before 2. prohphylaxis	after 2. prophylaxis	after 2. prophylaxis	after SRP
*S. oralis*	number	62/76	70/76	52/64	54/64	55/66	49/66	59/64	54/64	48/58	44/58
	%	81.6	92.1	81.3	84.4	83.3	74.2	92.2	84.4	82.8	75.9
	p value	0.055		0.639		0.201		0.169		0.359	
*S. mitis*	number	40/76	31/76	37/64	23/64	36/66	8/66	28/64	23/64	23/58	6/58
	%	52.6	40.8	57.8	35.9	54.5	12.1	43.8	35.9	39.7	10.3
	p value	0.143		**0.013**		**<0.001**		0.367		**<0.001**	
*S. gordonii*	number	6/76	14/76	6/64	9/64	6/66	14/66	13/64	9/64	9/58	12/58
	%	7.9	18.4	9.4	14.1	9.1	21.2	20.3	14.1	15.5	20.7
	p value	0.055		0.410		0.052		0.349		0.469	
*S.para sanguinis*	number	11/76	8/76	9/64	5/64	11/66	12/66	8/64	5/64	5/58	10/58
	%	14.5	10.5	14.1	7.8	16.7	18.2	12.5	7.8	8.6	17.2
	p value	0.462		0.257		0.819		0.380		0.166	
*S. sanguinis*	number	11/76	10/76	11/64	10/64	8/66	9/66	10/64	9/64	9/58	8/58
	%	14.5	13.2	14.5	13.2	12.1	13.6	15.6	14.1	15.5	13.8
	p value	0.814		0.811		0.795		0.804		0.793	
*S. intermedius*	number			2/64	1/64	2/66	4/66			1/58	4/58
	%			3.1	1.6	3.0	6.1			1.7	6.9
	p value			1.000		0.680				0.364	
*S. angi nosus*	number	15/76	9/76	15/64	3/64	14/66	5/66	9/64	3/64	2/58	3/58
	%	19.7	11.8	23.4	4.7	21.2	7.6	14.1	4.7	3.4	5.2
	p value	0.274		**0.002**		**0.026**		0.069		1.000	
*S. con Stellatus*	numberl	4/76	6/76	3/64	1/64	4/66	4/66	6/64	1/64	1/58	4/58
	%	5.3	7.9	4.7	1.6	6.1	6.1	9.4	1.6	1.7	6.9
	p value	0.513		0.619		1.000		0.115		0.364	
*S. acido Minimus*	number	3/76	2/76	3/64	2/64			2/64	2/64		
	%	3.9	2.6	4.7	3.1			3.1			
	p value	1.000		1.000				1.000			
*S. vesti Bularis*	number	3/76	3/76			2/66	2/66				
	%	3.9	3.9			3.0	3.0				
	p value	1.000				1.000					
*S. saliv. salivarius*	number	1/76	1/76	1/64	1/64	1/66	1/66	1/64	1/64	1/58	1/58
	%	1.3	1.3	1.6	1.6	1.5	1.5	1.6	1.6	1.7	1.7
	p value	1.000		1.000		1.000		1.000		1.000	
*S. saliv. thermophilus*	number	2/76	2/76	1/64	1/64	2/66	2/66	2/64	1/64	1/58	1/58
	%	2.6	2.6	1.6	1.6	3.0	3.0	3.1	1.6	1.7	1.7
	p value	1.000		1.000		1.000		1.000		1.000	
*S. dow nei/sobrinus*	number	3/76	1/76	3/64	3/64	3/66	2/66			3/58	1/58
	%	3.9	1.3	4.7	4.7	4.5	3.0			5.2	1.7
	p value	0.620		1.000		1.000				0.618	

**Table 4:** Prevalence of streptococci groups at different appointments and statistical analysis

groups of Streptococci		before 1. prophylaxis	before 2. prophylaxis	before 1. prophylaxis	after 2. prophylaxis	before 1. prophylaxis	after SRP	before 2. prophylaxis	after 2. prophylaxis	after 2. prophylaxis	after SRP
Mitis group	number	72/76	72/76	61/64	60/64	63/66	52/66	61/64	60/64	54/58	46/58
	%	94.7	94.7	95.3	93.8	95.5	78.8	95.3	93.8	93.1	79.3
	p value	1.000		1.000		**0.004**		1.000		**0.031**	
Sanguinis group	number	24/76	28/76	22/64	16/64	21/66	26/66	27/64	16/64	16/58	23/58
	%	31.6	36.8	34.4	25.0	31.8	39.4	42.2	25.0	27.6	39.7
	p value	0.494		0.246		0.363		**0.040**		0.169	
Anginosus group	number	18/76	14/76	17/64	5/64	17/66	12/66	14/64	5/64	4/58	10/58
	%	23.7	18.4	26.6	7.8	25.8	18.2	21.9	7.8	6.9	17.2
	p value	0.426		**0.005**		0.293		**0.025**		0.087	
Salivarius group	number	6/76	6/76	2/64	2/64	5/66	5/66	6/64	2/64	2/58	4/58
	%	7.9	7.9	3.1	3.1	7.6	7.6	9.4	3.1	3.4	6.9
	p value	1.000		1.000		1.000		0.273		0.679	

Considering just the groups of streptococci, statistically significant changes were found in the mitis, sanguinis, and anginosus groups. Positive results for the mitis group were reduced from 63 to 52 before the first appointment of professional prophylaxis and after SRP (p = 0.004). The difference between before and after SRP was also statistically significant (p = 0.031). The results for the sanguinis group were significantly different between before and after the second appointment of professional prophylaxis (p = 0.040), the positive results were reduced from 27 to 16. In the anginosus group the positive results were significantly reduced from 17 to five (p = 0.005) after two sessions of professional prophylaxis. The difference between, before, and after the second appointment of professional prophylaxis was also statistically significant (from 14 to five positive results, p = 0.025). No statistically significant changes over time were found for the mutans-group.

Just before the treatment the presence of supragingival plaque had an influence on the prevalence of several groups of streptococci. The mitis, sanguinis and salivarius groups were more often present in biofilm-positive sites (p = 0.016–0.036). The results are presented in Table 5.

**Table 5:** Prevalence of streptococci in the supragingival biofilm before treatment and statistical analysis

Groups of streptococci	Groups of interproximal supragingival biofilm (API)				Total	P value
	< 25%	≥ 25 % – < 42%	> 42–70%	>70%		
Mitis group						
n/N	12/12	7/8	13/16	40/40	72/76	0.016
%	100	87.5	81.3	100	94.7	
Sanguinis group						
n/N	2/12	0/8	4/16	18/40	24/76	0.036
%	16.7	0	25	45	31.6	
Anginosus group						
n/N	3/12	1/8	2/16	12/40	18/76	0.556
%	25	12.5	12.5	30	23.7	
Salivarius group						
n/N	0/12	1/8	4/16	1/40	6/76	0.027
%	0	12.5	25	2.5	7.9	

n = number of streptococci groups within the API-group; N = number of teeth within the API-group.

We found no statistically significant influence of the PD on the distribution of streptococci species. Only *S. parasanguinis* was found more often in pockets ≥ 6 mm (p = 0.036, data not shown).

## Discussion

SRP is the causative anti-infective and anti-inflammatory treatment procedure in the treatment phase of initial therapy of periodontitis.^32,35^ To maintain the achieved improvements continuous accurate mechanical plaque control should be performed by the periodontitis patient, which could be trained during anterior appointments of professional prophylaxis. It is of interest if the appointments of professional prophylaxis and the SRP have an influence on the amount of different streptococci species or groups because some of them are related to the carious process. It is worth to look if the change of the composition of the subgingival biofilm during the initial therapy is not only beneficial from the viewpoint of periodontitis but also for general health. This point is of interest because the presence of any microbial species is a point of interaction between the host and microorganism.

*S. mitis* was found more abundant on titanium surfaces in periodontally healthy patients.^31^ One may speculate that *S. mitis* is in relation to periodontal health. This is probable because Porphyromonas gingivalis as a strong periopathogenic bacteria and involved in the inflammatory process of periodontitis induces the cell death of *S. mitis* in an in vitro model.^6,14,38^ On the other hand this explanation does not totally fit with our results because after the SRP *S. mitis* is significantly reduced in our study. On the other hand Teles et al found a rapid recolonisation of supra- and subgingival biofilm compartments after professional tooth cleaning by *S. mitis* within one day already.^39^ One may speculate that the reduction of *S. mitis* due to the treatment procedures in our study is of minor importance for the composition of the subgingival biofilm because this species seems to recover very fast. *S. mitis* could be of higher interest in the supragingival root surface area because this species is considered as an early coloniser on the root surface as on the enamel and could promote the development of root caries.^13^

*S. anginosus* has been found in lower amounts in the subgingival biofilm when higher bacterial counts of *Campylobacter rectus* and *P. gingivalis* are present. These results were stated for untreated periodontitis patients with inadequately controlled diabetes mellitus.^37^ Statistically significantly more bacterial counts of *S. anginosus* were found in gingivitis and periodontitis sites in individuals with chronic bowel disease than in generally healthy periodontitis patients with gingivitis and periodontitis sites.^8^ Other studies have shown that after SRP the levels of *S. anginosus* have been also significantly reduced.^3,20^ From the point of view of dentinal caries the statistically significant reduction of *S. anginosus* as occurred in our study could be of interest because *S. anginosus* has been detected in areas with about neutral pH of dentinal caries.^25^

*S. oralis* has been found in higher levels at sites of healthy implants than in healthy sites of natural teeth and has been detected at significantly higher levels in the subgingival plaque of periodontitis patients.^19^ *S. oralis* has been also more frequently detected in the subgingival biofilm of patients with Down’s syndrome.^24^ Thiha et al described low levels of *S. oralis* in the subgingival plaque as well as in the gingival tissues of periodontitis patients.^40^ All these findings show that the role of *S. oralis* in the periodontal disease is not fully understood. One may speculate that the moderate reduction of *S. oralis* after one appointment of professional prophylaxis in our study is due to the reduction of the total amount of biofilm after the treatment. On the other hand, taking into account the extended caries ecological hypothesis this could reduce, even temporarily, the risk of dental caries.^30^ The intended elevation of the level of *S. oralis* by administration of streptococci containing probiotics had no effect on the periodontal status.^27^

Recently it was found that *S. gordonii* could be implicated in the process of bone resorption as demonstrated in vitro for periapical lesions.^33^ The lipoproteins of *S. gordonii* induce IL-8-production by human PDL-cells and contribute to the inflammatory process.^26^ Several authors described *S. gordonii* as involved in the progression of periodontal destruction, it is found in increased levels in the subgingival biofilm of alcoholics and the reduction of *S. gordonii* contributes to the promotion of periodontal health.^2,5,41^ Insofar it is surprisingly to a certain amount and cannot fully explain why the treatment did not result in stronger reduction of the *S. gordonii* level in our study. Anyway, *S. gordonii* is seen in relation to non-carious healthy tooth surfaces, probably because of the produced alkaline environment.^1,17^ In this context it could be of interest that the reduction of the level or the prevalence of members of the sanguinis group (including *S. gordonii*) as shown in our study for the second appointment of professional prophylaxis could contribute to periodontal health. On the other hand, no statistically significant changes over time were found for the mutans-group in our study. One may conclude that no statistically significant influence on the cariogenic activity of the studied subgingival biofilm was observed.

There are only few data about the mitis group. The statistically significantly higher prevalence of members of the mitis group in biofilm-positive sites could be a sign of early recolonisation of the root surface. Benjasupattananan et al found this group of streptococci in a higher amount in relation to lower gingival inflammation and in a caries-prone group.^7^

A relationship between the presence of the anginosus group and refractory course of periodontal disease was described.^9^ The statistically significantly reduced level of streptococci of the anginosus group after the two appointments of professional prophylaxis reflects in our study the improvement of periodontal health. On the other hand, another member of the anginosus group, *S. constellatus*, is apparently a stronger periopathogenic bacteria.^10^

The study has some limitations since just one treatment arm has been followed-up and a reduced but sufficiently big enough number of patients with moderate to severe periodontitis has been studied by comprehensive microbiological laboratory work. From this study one may conclude that step-wise periodontal therapy influences several streptococci like *S. mitis* and *S. anginosus*. On the other hand this study also shows that no tremendous increase of specific streptococci especially related to the carious process in the subgingival biofilm occurs.

## References

[ref1] Abranches J, Zeng L, Kajfasz JK, Palmer SR, Chakraborty B, Wen ZT (2018). Biology of Oral Streptococci. Microbiol Spectr.

[ref2] Amaral Cda S, da Silva-Boghossian CM, Leão AT, Colombo AP (2011). Evaluation of the subgingival microbiota of alcoholic and non-alcoholic individuals. Dent.

[ref3] Artese HP, de Sousa CO, Torres MC, Silva-Boghossian CM, Colombo AP (2012). Effect of non-surgical periodontal treatment on the subgingival microbiota of patients with chronic kidney disease. Braz Oral Res.

[ref4] Banas JA, Drake DR (2018). Are the mutans streptococci still considered relevant to understanding the microbial etiology of dental caries?. BMC Oral Health.

[ref5] Bascones A, Gamonal J, Gomez M, Silva A, Gonzalez MA (2004). New knowledge of the pathogenesis of periodontal disease. Quintessence Int.

[ref6] Bender P, Egger A, Westermann M, Taudte N, Sculean A, Potempa J (2018). Expression of human and *Porphyromonas gingivalis* glutaminyl cyclases in periodontitis and rheumatoid arthritis-A pilot study. Arch Oral Biol.

[ref7] Benjasupattananan S, Lai CS, Persson GR, Pjetursson BE, Lang NP (2005). Effect of a stannous fluoride dentifrice on the sulcular microbiota: a prospective cohort study in subjects with various levels of periodontal inflammation. Oral Health Prev Dent.

[ref8] Brito F, Zaltman C, Carvalho AT, Fischer RG, Persson R, Gustafsson A (2013). Subgingival microflora in inflammatory bowel disease patients with untreated periodontitis. Eur J Gastroenterol Hepatol.

[ref9] Colombo AP, Haffajee AD, Dewhirst FE, Paster BJ, Smith CM, Cugini MA (1998). Clinical and microbiological features of refractory periodontitis subjects. J Clin Periodontol.

[ref10] Colombo AP, Bennet S, Cotton SL, Goodson JM, Kent R, Haffajee AD (2012). Impact of periodontal therapy on the subgingival microbiota of severe periodontitis: comparison between good responders and individuals with refractory periodontitis using the human oral microbe identification microarray. J Periodontol.

[ref11] Darveau RP (2010). Periodontitis: a polymicrobial disruption of host homeostasis. Nat Rev Microbiol.

[ref12] Darveau RP, Hajishengallis G, Curtis MA (2012). Porphyromonas gingivalis as a potential community activist for disease. J Dent Res.

[ref13] Do T, Damé-Teixeira N, Naginyte M, Marsh PD (2017). Root surface biofilms and caries. Monogr Oral Sci.

[ref14] Duran-Pinedo AE, Baker VD, Frias-Lopez J (2014). The periodontal pathogen Porphyromonas gingivalis induces expression of transposases and cell death of Streptococcus mitis in a biofilm model. Infect Immun.

[ref15] Eick S, Mathey A, Vollroth K, Kramesberger M, Bürgin W, Sculean A (2017). Persistence of *Porphyromonas gingivalis* is a negative predictor in patients with moderate to severe periodontitis after nonsurgical periodontal therapy. Clin Oral Investig.

[ref16] Facklam R (2002). What happened to the streptococci: overview of taxonomic and nomenclature changes. Clin Microbiol Rev.

[ref17] Fakhruddin KS, Ngo HC, Samaranayake LP (2018). Cariogenic microbiome and microbiota of the early primary dentition: a contemporary overview. Oral Dis.

[ref18] Fernando S, Kumar S, Bakr M, Speicher D, Lea R, Scuffham PA (2018). Children’s untreated decay is positively associated with past caries experience and with current salivary loads of mutans Streptococci; negatively with self-reported maternal iron supplements during pregnancy: a multifactorial analysis. J Public Health Dent.

[ref19] Gürlek Ö, Gümüş P, Nile CJ, Lappin DF, Buduneli N (2017). Biomarkers and bacteria around implants and natural teeth in the same individuals. J Periodontol.

[ref20] Haffajee AD, Uzel NG, Arguello EI, Torresyap G, Guerrero DM, Socransky SS (2004). Clinical and microbiological changes associated with the use of combined antimicrobial therapies to treat ‘refractory’ periodontitis. J Clin Periodontol.

[ref21] Hajishengallis G, Darveau RP, Curtis MA (2012). The keystone-pathogen hypothesis. Nat Rev Microbiol.

[ref22] Invernici MM, Salvador SL, Silva PHF, Soares MSM, Casarin R, Palioto DB (2018). Effects of Bifidobacterium probiotic on the treatment of chronic periodontitis: a randomized clinical trial. J Clin Periodontol.

[ref23] Jones AA, Kornman KS, Newbold DA, Manwell MA (1994). Clinical and microbiological effects of controlled-release locally delivered minocycline in periodontitis. J Periodontol.

[ref24] Khocht A, Yaskell T, Janal M, Turner BF, Rams TE, Haffajee AD (2012). Subgingival microbiota in adult Down syndrome periodontitis. J Periodontal Res.

[ref25] Kianoush N, Adler CJ, Nguyen KA, Browne GV, Simonian M, Hunter N (2014). Bacterial profile of dentine caries and the impact of pH on bacterial population diversity. PLoS One.

[ref26] Kim AR, Ahn KB, Kim HY, Seo HS, Kum KY, Yun CH (2017). *Streptococcus gordonii* lipoproteins induce IL-8 in human periodontal ligament cells. Mol Immunol.

[ref27] Laleman I, Yilmaz E, Ozcelik O, Haytac C, Pauwels M, Herrero ER (2015). The effect of a streptococci containing probiotic in periodontal therapy: a randomized controlled trial. J Clin Periodontol.

[ref28] Lang NP, Bartold PM (2018). Periodontal health. J Clin Periodontol.

[ref29] López R, Dahlén G, Retamales C, Baelum V (2011). Clustering of subgingival microbial species in adolescents with periodontitis. Eur J Oral Sci.

[ref30] Marsh PD, Head DA, Devine DA (2015). Ecological approaches to oral biofilms: control without killing. Caries Res.

[ref31] Martínez-Hernández M, Olivares-Navarrete R, Almaguer-Flores A (2016). Influence of the periodontal status on the initial-biofilm formation on titanium surfaces. Clin Implant Dent Relat Res.

[ref32] Ottawa (ON) (2016). Dental scaling and root planing for periodontal health: a review of the clinical effectiveness, cost-effectiveness, and guidelines [Internet]. Canadian Agency for Drugs and Technologies in Health.

[ref33] Park OJ, Kim J, Kim HY, Kwon Y, Yun CH, Han SH (2018). *Streptococcus gordonii* induces bone resorption by increasing osteoclast differentiation and reducing osteoblast differentiation. Microb Pathog.

[ref34] Rupf S, Brader I, Vonderlind D, Kannengiesser S, Eschrich K, Roeder I (2005). In vitro, clinical, and microbiological evaluation of a linear oscillating device for scaling and root planing. J Periodontol.

[ref35] Sanz I, Alonso B, Carasol M, Herrera D, Sanz M (2012). Nonsurgical treatment of periodontitis. J Evid Based Dent Pract.

[ref36] Sanz M, Beighton D, Curtis MA, Cury JA, Dige I, Dommisch H (2017). Role of microbial biofilms in the maintenance of oral health and in the development of dental caries and periodontal diseases. Consensus report of group 1 of the Joint EFP/ORCA workshop on the boundaries between caries and periodontal disease. J Clin Periodontol.

[ref37] Silva-Boghossian CM, Orrico SR, Gonçalves D, Correa FO, Colombo AP (2014). Microbiological changes after periodontal therapy in diabetic patients with inadequate metabolic control. Braz Oral Res.

[ref38] Tada H, Nishioka T, Takase A, Numazaki K, Bando K, Matsushita K (2018). *Porphyromonas gingivalis* induces the production of IL-31 by human mast cells, resulting in dysfunction of the gingival epithelial barrier. Cell Microbiol.

[ref39] Teles FR, Teles RP, Uzel NG, Song XQ, Torresyap G, Socransky SS (2012). Early microbial succession in redeveloping dental biofilms in periodontal health and disease. J Periodontal Res.

[ref40] Thiha K, Takeuchi Y, Umeda M, Huang Y, Ohnishi M, Ishikawa I (2007). Identification of periodontopathic bacteria in gingival tissue of Japanese periodontitis patients. Oral Microbiol Immunol.

[ref41] Wang H, Ai L, Zhang Y, Cheng J, Yu H, Li C The effects of antimicrobial peptide Nal-P-113 on inhibiting periodontal pathogens and improving periodontal status. Biomed Res Int.

[ref42] Ximénez-Fyvie LA, Haffajee AD, Som S, Thompson M, Torresyap G, Socransky SS (2000). The effect of repeated professional supragingival plaque removal on the composition of the supra- and subgingival microbiota. Clin Periodontol.

